# 'Compassion, the first emotion ditched when I’m busy’. The struggle to maintain our common humanity

**DOI:** 10.15694/mep.2018.0000167.1

**Published:** 2018-08-14

**Authors:** Lorna Davin, Jill Thistlethwaite, Emma Bartle

**Affiliations:** 1 University of Notre Dame; 2 University of Technology; 3 University of Queensland

**Keywords:** Compassion, humanism, our common humanity, narrative, doctor identity, emotion, safety ethic, prosocial moral emotion, self-compassion

## Abstract

This article was migrated. The article was marked as recommended.

Introduction

A considerable body of literature has been built around the socialisation of medical students and junior doctors into the culture of medicine, yet our appreciation of how their affective learning is shaped through practice, over time, continues to challenge our understanding and subsequent educational practice. This study addresses this gap by using compassion as a lens to unpack affective learning.

Methods

This research asked interns undertaking their first year of medical practice “What have been the main influences (positive and/or negative) in how you have learned to express compassion for your patients when working in the clinical context?” Their individual narratives, generated through reflective journals and unstructured interviews, when thematically analysed, told us how and why they struggled.

Findings

The eight interns expressed their struggle to maintain their compassionate aspirations when confronted with the complexity and competing demands of their community of practice. Their emotional disquiet triggered their safety ethic resulting in their compassion, a prosocial moral emotion, being replaced by a more reductionist approach where patient care was reframed as patient management.

Discussion

While neither inevitable nor static, the interns’ narratives tell a story where, after a year embedded in their community of practice, their increased self-efficacy, derived primarily from their perceived biomedical competence, enables them to revisit their original aspirations - to be both compassionate and competent - recognising that being a ‘good’ doctor does not have to eclipse being a kind and caring human being.

Conclusions

The interns’ reflections uncovered a narrative of emotional vulnerability, where fearing failure and seeking perfection, contributed to a diminished self‑efficacy resulting in risk aversive behaviours protecting their doctor identity. In the recommendations the authors propose strategies for safe engaged connection, where self‑understanding replaces self‑criticism and self‑compassion is cultivated to guard against contempt and cynicism.

## Introduction

A considerable body of literature has been built around the socialisation of medical students and junior doctors into the culture of medicine. A continuous theme throughout this literature on professional acculturation is the interplay between the various curricula: the perceived disconnect between the formal, informal, hidden and the null curricula ( Hafferty and Hafler, 2011), ( Hundert, Hafferty and Christakis, 1996), ( Karnieli-Miller *et al.*, 2010), ( Shapiro *et al.*, 2009), ( Wear and Castellani, 2000), Research continues to highlight the contradictions inherent in how the medical curriculum is framed with ‘explicit commitment to traditional values of doctoring - empathy, compassion and altruism among them - and a tacit commitment to behaviours grounded in an ethic of detachment, self‑interest and objectivity’ ( Coulehan and Williams, 2001).

Interns, as novice doctors are greatly influenced by the culture of medicine and the implied messages conveyed through role models, expectations, informal conversations, overt behaviours, and social norms. The doctors construct meaning from those influences which may then influence their future practice ( Kumagai, 2008).

An editorial in the Lancet (2007) laments that, ‘although compassion is often cited as one of the core values of professionalism, there remains a continuous and inconclusive debate about whether compassion is innate or whether it can be taught?’. However, Treadway and Chatterjee (2011) suggest we are asking the wrong question. They contend that most students come to medicine caring, but through neglect and silence, they are taught not to care. They conclude that the focus of medical education and training should be on the ‘how’ of caring. The Lancet (2007) editorial concludes ‘to make care more than a manufactured product there also needs to be compassion - the ability to feel for someone in trouble’. This study explores the nature of compassionate care, a core concept which weaves its way through medical literature on professionalism and humanism ( Back *et al.*, 2009), ( Bonic, 2004), ( Burnell, 2009), ( Cooney, 2005), ( Gelhaus, 2013), ( LeBaron, 2004), ( Lown, Rosen and Marttila, 2011), ( Marcum, 2011), ( McClenon, 1996), ( Redelmeier, Molin and Tibshirani, 1995), ( Wiggins, Coker and Hicks, 2009) asking interns to share how they have learned to express compassion for patients when working in the clinical context.

This study specifically responds to this gap in the literature. Medical educators need to have a deeper, nuanced understanding of how the affective aspects of caring, are learned through practice. Through asking fledgling doctors to share their experiences regarding their expression of compassion, to build our understanding as educators, we can then work to ensure these learning opportunities continue to be nurtured and valued.

## Methods

### The Participants, Participating University and Teaching Hospitals

The University of Queensland, School of Medicine (UQ SOM) Australia, offers a four year graduate entry program across several campuses.

An email invited all 401 Year 4, UQ SOM students to participate in the following reflection, ‘What have been the main influences (positive and/or negative) in how you have learned to express compassion for your patients when working in the clinical context?’ The fifty six students who chose to participate were invited to continue in a longitudinal prospective study as they transitioned to their intern year. Eight of the fifty six consented to continue in the longitudinal study while working in eight different teaching hospitals in Australia - their narratives are the focus of this paper.

### Methodology

Education as a field of study does not lend itself easily to a reductive research paradigm, with the range of confounding variables creating difficulty in transferring outcomes to broader populations ( Mann, 2004), ( Mann, 2011), ( Wong et al., 2012). When studying affect, these issues are amplified as the very definitions of attitudes, values, beliefs and emotion are open to interpretation. Interpreting the complex narratives generated by the participating interns over the year was complicated. Interpretation through a single theoretical lens appeared inadequate, an artificial fit of their stories to an imperfect paradigm.For the purpose of this study, in analysing the narrative of the interns we have interpreted their individual meaning making broadly, using Bandura’s self-efficacy theory ( Bandura, 1994). Self-efficacy theory is a constructivist theory which allows us to better understand and interpret the individual meaning-making related to the sociocultural influences of the learning environment through foregrounding the individual.

These influences are inextricably linked with the development of the novice doctor’s fledgling professional identity within their community of practice (COP) ( Wenger, 2007) which belongs to the constructionist theoretical perspective. Alongside practice and community, identity is an integral aspect of Wenger’s COP theory. ‘Identity serves as a pivot between the social and the individual .. it avoids the simplistic individual - social dichotomy’ ( Wenger, 2007, p. 145). It is the interplay between the person and the community that has primacy in the development of identity: ‘we cannot become human by ourselves’ ( Wenger, 2007, p. 146).In interpreting the interns’ individual narratives over the year, Wenger’s COP theory is a useful approach to follow their temporal journey as they transition from peripheral to fuller practice. These individual, detailed narratives are reported elsewhere. ( Davin, 2016).

This non-binary positioning of the theoretical approach allows the interpretive lens to move back and forth reframing the exploratory nature of this study, embracing individual agency within the collective sociocultural influences of learning through practice. While some would argue the theoretical perspectives are incompatible, Kohler Reissman (2003, p. 23) posits in her re-interpretation of her own identity and illness narratives, ‘understanding complex lives requires more than one theoretical lens’, and so too for this research.

Central to this interpretive approach is the understanding that medical education is no longer only about the acquisition of siloed knowledge, skills and attitudes; it is about the construction of a professional identity ( Mann, 2004), ( Rees and Monrouxe, 2010), ( Reeves et al., 2008), ( Swanwick, 2005).

### Methods and Data Collection

### Journaling and in-depth unstructured interviews

Anchored in the overarching research question, ‘‘What have been the main influences (positive and/or negative) in how interns learn to express compassion for patients when working in the clinical context?’’ The interns were asked to reflect on their intern year, (a conditional year of practice where they are assessed across rotations in emergency and general medicine, surgery and other rotations) by journaling any relevant experiences which they found meaningful in their day to day clinical practice. Beyond requesting they record their experiences, no other specific directions were given. Participants were free to choose how they would record their journal. They used a range of media including typed electronic journals, blogs, video and audio journals which they returned by email. Participants also chose how often, and how much, they shared. If necessary, the participants received reminders about their journal by email as a prompt, two weeks prior to their interview, inviting them to contribute.The interns also participated in three interviews every four months over 12 months. The data provided by the interns’ journals were used to facilitate the more in-depth discussion in the unstructured interviews. Psuedonyms have been used to protect the identity of the eight interns.

### Thematic analysis

In keeping with the interpretive nature of this study, drawing on existing literature the individual narratives of the interns, taken collectively, were thematically analysed informed by broad guidelines developed by Braun and Clarke (2006), and specific constructs defined by Schultz (1970) and further interpreted by Titchen and McIntyre (1993). Within this framework, first order constructs are those provided by the research participant and second order constructs are those defined by the researchers ( Schutz, 1970), ( Titchen and McIntyre, 1993). To enable the students to construct their own meaningful understanding of compassion their reflections were not limited by a specific definition provided by the researchers.

## Results/Analysis

The themes which shed light on and build understanding of the novice doctors learning to be compassionate, when engaged in a complex community of practice, are listed in Table 1 and discussed below:

**Table 1.** List of identified themes

Identified Themes
Ill‑prepared and overwhelmed
Pursuing Perfection and Fearing Failure
See‑Sawing Self-efficacy
Distancing emotion
Transcending doctor identity - common humanity

### Ill‑prepared and overwhelmed

*I’ve started my internship in Emergency. The first two weeks were so overwhelming, it was like being a medical student with too much responsibility.. I wanted to cry at some point during every one of these shifts and was adamant (sic) it was unfair to throw us in the deep end like this..*

*(Grace)*

Despite being exposed to clinical practice during rotations as medical students, a key theme highly influential in shaping the learning trajectory and practice of each of the participating interns, was the way in which they struggled with the entry to their internship. While being ill‑prepared is a familiar trope in the literature ( Ackerman *et al.*, 2009), ( Brady, Corbie-Smith and Branch, 2002), ( Kilminster *et al.*, 2011), central to this interpretation of their transition in the context of compassionate care is their emotional response to their new role. The enormity of the responsibility required of the interns initially took them by surprise, resulting in emotional turmoil and vulnerabilty. They were scared, shocked and overwhelmed by their experiences during this daunting transitional period.

### Pursuing Perfection and Fearing Failure

*How can you transcend to be perfect and compassionate and empathetic clinician when you don’t know where you stand?*

*(Trevor)*

A significant theme interpreted through the interns’ narrative is the idealised notion of perfection. Wanting to be a ‘good’ doctor quickly morphed into aspiring to perfection. Perfection, in this context, is a complex construct resulting in narrow, risk aversive behaviours as they attempt to grasp for increased control over their work environment, all ostensibly linked to the fear of failure. Failure to fulfil the requirements of the good doctor identity enmeshed in their clinical role: an identity framed by the interns’ unmet expectations and aspirations based on an idealised notion of perfection - the perfect doctor, with perfect knowledge administering care to the perfect patient - compounded by the overwhelming sense of uncertainty they confronted in undertaking the responsibilities of their role in a messy and complex workplace.

### See Sawing Self-efficacy

The interns’ self‑efficacy had a major influence on how they delivered care. Their own perceived success or failures derived primarily from biomedical competence, in addition to the consequences of observing their peers and colleagues as both positive and negative role models - contributed to how they learned to care. Feedback from peers, senior colleagues and patients was central to their ongoing confidence and competence creating a seesawing effect, where positive or negative feedback created an emotional rollercoaster heightening their vulnerability.

Within the interns’ narrative it is evident that their self‑efficacy, how they perceive they are performing in their prescribed role, ( Bandura, 1994) is shaped both implicitly and explicitly.

*Certain stressors tend to affect my mindset such as workload and support from the senior doctors. When the senior doctors are critical then it affects my confidence in being able to adequately care for patients, and this in turn makes me feel like a fraud in front of my patients and as if I do not have the right to be caring for them and showing compassion. The reverse of this is that when I feel like I am doing well at my job I find it very easy to have compassion.*

*(Nathan)*

Their emotional response, both embodied, (expressed physically, for eg. wanting to vomit), and visceral, (expressed emotionally for eg. in wanting to cry), sat uncomfortably with the professional identity they wished to create for themselves as a doctor in competently meeting their patients’ needs and conforming to cultural norms of their profession.

### Distancing Emotion

Reflecting the unwritten rules of their community of practice, the hidden curriculum, ‑ where emotion is either noticeably absent ( McNaughton, 2013) or continues to be reframed as detached concern ( Coulehan and Williams, 2001), the interns preferred to ‘act on the side of coldness’ (Intern Nathan) rather than to be seen as too emotional.

Their journal entries are replete with their emotional responses in wanting to cry, and/or vomit, feeling sad, putting their emotions on hold (e.g. going home to cry), while simultaneously drawing boundaries around the emotional needs of their patients by not engaging too closely.

*I hate it and I’m counting down the days. This is also mainly about working with families but it is within a geriatric population. They all remind me of my own grandparents and it pulls on my heart strings. I do a lot of ‘Not for resuscitation’ orders, death certificates and family meetings. I almost find it bizarre how removed I am from it, I don’t know if it is a lack of interest in the area or just a protective mechanism.*

*(Grace)*

This triggered a range of protective behaviours as they built a protective armoury, erecting barriers around emotion and patient engagement.

### The Triage of Compassionate Care

Alongside the expectations the interns created for themselves as doctors, they simultaneously held an idealised notion of the ‘perfect patient’. This idealised notion of the perfect patient, portrayed the patient as someone likeable, treatable and grateful: a romanticised representation far removed from the reality of day to day practice.

Contrasting with this idealised notion of the perfect patient was the patient whose behaviours and attributes were perceived by the interns to manifest in a range of negative ways directly influencing how they provided care.

In her journal entries, Mary describes her ‘worst patient’ as follows:

*Worst patient I’ve had is probably this drunk who threatened to rape the female staff and swore and threw punches and refused treatment..I handed him over to another intern, a male one because like hell I was going to stay around to be abused like that.*

*(Mary)*

A constant thread recurring through the interns’ reflections is the belief that, if the patient’s behaviour is perceived to be inappropriate, presenting with what they perceive to be trivial problems, then they are less deserving of ‘extra’ care. Compassion appears to be an optional adjunct to their role with medical knowledge, clinical reasoning and procedural skills determining their core competencies as a ‘good’ doctor.

### Transcending the Doctor Identity - Common Humanity

Most significantly, as the interns completed their full, year-long internship, a shift in their thinking, attitudes and behaviour became evident. There was evidence of resilience and empowerment which enabled them to reconnect with their original intent to be a compassionate practitioner.

Once they felt confident and competent and, as they incrementally became more comfortable with their role and identity as a doctor, they were afforded the time to be more caring for their patients. They referred to ‘ *the little things*’ - finding a blanket, making sure the patient is not hungry or thirsty - little things - which you do not need to be a doctor to do. These ‘little things’ which they consider ‘not doing anything’ - the very ‘things’ as illustrated by Grace in her journal, perceived as so meaningful to the patient as an expression of care:

*There’s heaps of anti‑emetics [for vomiting] and analgesics to offer patients and besides that a bottle of water or an extra warm blanket never goes astray.*

*This term has probably taught me a lot about doing the things that make people feel cared for even if it’s not the things that make you feel that you are caring for them. To me, caring for someone is giving them a diagnosis, a prognosis or a solution. For them it seems that it’s more about the little things. Whether that’s because patients associate caring about the small things with caring about the big things or because patients in pain care more about their sore foot than their heart failure that is causing it I don’t know. Either way, I’ll be offering more bottles of water from now on’.*

*(Grace)*

For Mary, her final journal entry illustrates how the everyday demands of the role continued to intrude on her desire to be compassionate. As she finished the year she reflected on the relevance and meaning of caring in a compassionate way:

*Compassion tends to be the first emotion ditched when I’m busy. Intellectually, I know I should care about what the patients and their families are going through but it’s just easier not to because there’s no time. Got to get that cannula in. Get those bloods sent off. Get the referral done. Get imaging forms in. I was filling out the care of the dying pathway form and I realised I hadn’t even SPOKEN to the patient or his family. But I just didn’t care. Until now ..*

*(Mary)*

In his final journal entry for the year, Nathan captures the essence of many of the inter‑related themes referred to across the interns’ narrative and subsequent analysis. He writes how, initially focused on developing his identity as a doctor, he crafted a professional persona which created boundaries and a power differential within the ‘doctor patient relationship’, emphasising difference rather than sameness - a relationship he now challenges:

*After a year of working I feel I have reached a conclusion regarding compassion. The term ‘doctor‑patient relationship’ is a term which reminds us to consider themes like duty, confidentiality, boundaries, power differential and illness. But I feel this term detracts from the real relationship which is a human‑human interaction.*

*(Nathan)*

## Discussion

In drawing together commonalities across the individual narratives, the thematic analysis, as a collective narrative, illuminates for the reader the complex interplay between the novice doctors’ expectations and aspirations as it unfolds.

No longer a student on the periphery, loosely attached or tethered to a team, the interns grapple with being a doctor - with all the incumbent expectations of the role. There is no slow unfolding of increased responsibility as they shift from the periphery of practice to fuller responsibility within their community of practice ( Wenger, 2007). Despite holding the title doctor, acceptance and recognition remain conditional; it is a role underpinned by responsibility with expected knowledge, skills and behaviour dependent on engagement across the team.

Their collective voice tells a story of the competing tensions which confront them. Aspiring to the idealised notion of the perfect doctor, when feeling emotionally overwhelmed, uncertain, imperfect ‑ fearing failure ‑ and confronted with competing priorities, the interns initially compromise; reframing their role to a more reductionist function focused on biomedical competence.

Struggling with their own and their patients’ emotional demands, they develop protective barriers, creating a distance between themselves and their patients, while aligning themselves with their colleagues. Self‑efficacy, their self‑perception of how well they are achieving in their role, is shaped by several key factors. According to Bandura (2001, p. 10), self‑efficacy is a powerful determinant of behaviour as it influences ‘what challenges to undertake, how much effort to expend in the endeavour, how long to persevere in the face of obstacles and failures, and whether failures are motivating or demoralizing’. For the interns in this study, their own perceived success or failure in expressing compassion is a major enabler or inhibitor in how they learn to express compassionate care in their developing role. The observation of other doctors as highly influential role models, in their practice of compassion, or their lack of compassion in practice, is also a significant influence. The consequences of their own, and or others behaviours, either negative or positive, alongside the feedback they receive from others were major influences on how they felt and how they acted towards their patients.

Foregrounding the individual’s cognition, self‑efficacy provides us with a window of understanding into the interplay between emotion and cognition in constructing a doctor’s identity. For the interns, sociocultural expectations and the relational connection and engagement with other people, provide a feedback loop which shapes the way in which the doctor expresses compassion. de Zulueta (2013) writes about a similar mechanism, describing how when survival (in the context of this study, the interns’ identity as a doctor) is under threat we narrow our focus resulting in a diminished ability to be kind and caring (de Zulueta, 2013). In exploring similar concepts in the context of school students, Zakrzewski (2015) suggests we cannot cultivate emotional intelligence in the absence of a moral rudder. She suggests that in order to cultivate a pro‑social ethic for ourselves and others we need to encourage self‑reflection and self‑awareness ( Zakrzewski, 2015).

The passage of time, and learning embedded in practice, is a major influence on how the doctors provide care. While neither inevitable nor static, the interns’ narratives tell a story where, after a year embedded in their community of practice, their increased self-efficacy, derived primarily from their perceived biomedical competence, enables them to revisit their original aspirations - to be both compassionate and competent - recognising that being a ‘good’ doctor does not have to eclipse being a kind and caring human being.

However, the doctor’s self‑efficacy is fragile. When challenged in practice, and not afforded the safety-net of supportive supervision, the doctor’s self-efficacy can be easily destabilised - analogous to an hourglass being inverted - where the natural flow of events is disrupted. The doctor’s thoughts, feelings and actions again become clouded. Feeling vulnerable, they readily default to the safety ethic of self-protection. However, when equilibrium returns, and *if supported*, they regain their desire to be compassionate.

Their ability to return to their original aspiration to be a compassionate, caring doctor is dependent on both self‑awareness and self‑understanding. Without understanding the nature of these influential dynamics, interns may remain captive to a repetitive and destructive cycle of diminished self‑efficacy and self‑criticism, detrimental to both themselves and the patients for whom they care; a constant inverting of the hour‑glass where they become stuck in the default for survival. However, in general, the interns’ perceptions and practice shifted over time, illuminating a narrative of growth which builds our understanding of the influence of the enabling and inhibiting factors in how they learn to express compassion within their community of practice. Their narrative illustrates how a complex social construct ‑ identity - developed within their community of practice with all its incumbent societal expectations and individual aspirations, enmeshed with a fragile self‑efficacy, shapes the novice transitioning from the periphery of practice on an inbound and eventually insider trajectory.

The interns who participated in this study did so because compassion was an attribute they wished to retain and develop, as core to their personal and professional practice. However, while being compassionate was an attribute to which they aspired, their intentions initially became derailed. Their nuanced and complicated behaviours and narrated experiences did not fit neatly into discrete, linear categories. As young people, their accumulative, transformative experiences created fissures in their worlds, as they confronted death, disease and patient misery head‑on.

In completing the narrative arc, the interns have adapted, compromised and at times conformed; navigating a complex myriad of clinical practice to the year’s end. No longer on the periphery, they have gained legitimate entry into their community of practice. Their emergent identity, while wavering at times, continues to extend to compassionate care, perhaps more pragmatic and more practical, but not lost to the demands of the complex system or medical culture in which they work and by which they are now defined.

### Limitations

No matter the efforts made to be inclusive in our research, some aspects of the interns’ learning experiences will have escaped the researchers’ gaze, and other aspects will have been looked upon with more intensity. There is neither objective nor true interpretation. Reflexivity provides a mechanism to make our interpretation transparent, not to create an objective certainty.

Having co-researchers provides an opportunity for alternative interpretations and questioning of assumptions. A participant validation of the narratives written for each of the interns was undertaken predominantly to ensure they were comfortable with the amount of information shared about them, rather than agreeing with the co-constructed narratives. Ultimately as a qualitative study, a singular truth is neither desirable nor possible. Each of us, the intern, the researchers and the reader, have brought our own perspectives to the reading which will frame our understandings; these too, may change over time, experience and audience.

Furthermore, the interns who participated in this study do not represent the full diversity of all novice doctors. They obviously valued compassion - yet they struggled. What then for those who do not identify with compassion as a humanistic practice of value for themselves or their patients - what of their stories? Exploring the narrative arc of those who chose not to participate could provide further insights into the expression of compassion through practice.

Being an interpretive study, the findings cannot be considered generalisable to all junior doctors. However, transferability of the findings to a similar cohort in a similar context may assist us as educators in our understanding, better scaffolding their learning and development as doctors and people.

## Conclusion

This study shines a light on the complex interplay between individual meaning making and the sociocultural context of practice. Captured in the novice doctors’ collective narrative and thematic analysis is a shared story which adds new understandings to the shaping of the expression of compassion, or at times, the development of cynicism. What was learned after a year embedded in practice was that the doctor‑patient relationship, above all else, was a human to human relationship, where boundaries transcend both the personal and professional.

What then does this mean for us as health professional educators, for our colleagues, our students, and for our patients - being each of us - as past, present or future consumers of healthcare? Our challenge as educators is to provide a ‘shelter of practice’ (Intern Trevor), through nurturing the individual learner, while cultivating a supportive, well supervised learning context and culture conducive to embracing compassion for self and other, which continues to be patient centred. We need to nurture a culture which gives voice to recognising and addressing the emotional aspects of caring while protecting both the doctor and patient’s ongoing emotional wellbeing.

### Recommendations

In Table 2, the authors propose recommendations and strategies for safe engaged connection, where self‑understanding replaces self‑criticism and self‑compassion is cultivated to guard against contempt and cynicism. The recommendations extend across the continuum from student, junior doctor to consultant and changes suggested can be understood and acted on through both individual agency and across teaching and learning within collective practice. They speak to both the ‘new comer’ and the ‘old timer’.

**Table 2.** Recommendations and Implications for Future Practice

Learning Experience	Recommendation and Implication
Transitioning to Practice	Prior to graduating, maximise every opportunity you have to spend time in the clinical environment. Spend time becoming familiar with the systems and processes. Despite being on the periphery of practice every observation should be considered a learning opportunity
Emotional Vulnerability	Recognise and accept your own vulnerability as a person. Reflect on how you judge yourself - your self-efficacy, self‑esteem, self‑worth. Develop a deliberate practice of self-awareness, self‑understanding, self‑compassion. Consider Neff’s (2011) work on the three pillars of self‑compassion ‑ kindness, mindfulness and our common humanity. Reflect, write, blog, participate and engage in a safe, confidential environment. Reflect on how to draw safe boundaries around emotion as an alternative to reacting with overly protective barriers Learn mindfulness through books, audio‑visuals, on‑line, classes or through one to one counselling. If you are struggling emotionally, self‑refer to the free, confidential counselling services provided by your employee or university. Be wary of identifying your clinical competence as a sole indicator of your worth as a doctor and as a person. Reflect on what frame of mind you default to, when stressed and emotionally vulnerable or overwhelmed. Extending Zakrzewski’s (2015) work, ask yourself: Am I rushing? How am I prioritising my time and competing demands? Do I feel the patient deserved their suffering? Am I overwhelmed by my own emotional situation, and/or the patients? Do I feel more powerful than the patient needing help? Do I feel safe in my community of practice? Pause and reflect on your thoughts and feelings and on how you can reframe your thinking, and who you can ask for support. Reflect on how you are judging yourself.
Identity	Embrace our common humanity, the notion of shared suffering. Recognise that suffering is a part of each of our lives, your patient may be suffering, but so too may you in having to respond to their needs. Embrace the notion of imperfection, your own, your patients and medicine as an imperfect science. Differentiate between uncertainty and inexperience Be aware of wanting to belong, ‘othering’ and the subsequent triage of compassionate care.
Feedback and Supervision	Actively communicate your learning and supervisory needs. Actively request feedback and support on what you are doing well and how you could improve. When you make a mistake or have a slip in your professionalism, be courageous enough to discuss what happened and why, in a safe place with safe people; support others when they do the same. Initiate feedback to peers and colleagues on what they have done well and how their practice could improve. Initiate safe discussions on how you and/or others feel emotionally when responding to difficult patient scenarios. Provide opportunities for staff to debrief either formally or informally. Give constructive feedback when someone has done well or tried and failed. Look for behaviours/systems/processes and communications that work well. Comment constructively and consider ways to extend their reach.

## Take Home Messages

• Be wary of over-identifying with your clinical competence as a sole indicator of your worth as a doctor and as a person
• Embrace our common humanity, the notion of shared suffering
• Embrace the notion of imperfection, your own, your patients and medicine as an imperfect science
• Reflect on how to draw safe boundaries around emotion as an alternative to reacting with overly protective barriers
• Do not allow attempting to be the *perfect* doctor eclipse being a kind and caring human being

## Notes On Contributors

Dr. Lorna Davin is a senior lecturer in Medical Education, at the University of Notre Dame Australia (UNDA), Fremantle. She is Program Coordinator for, and teaches into, the UNDA Health Professional Education suite of programs. Lorna has over 25 years’ experience in health professional education in both hospital and community settings.

Prof. Jill Thistlethwaite is a health professional education consultant, general practitioner, adjunct professor University Technology Sydney, honorary professor University of Queensland and medical adviser NPS MedicineWise, Sydney Australia. She is Editor-in-chief *The Clinical Teacher* and associate editor *Journal of Interprofessional Care.*

Dr. Emma Bartle is a lecturer at the School of Dentistry, University of Queensland. Her roles focus on building the teaching and learning capacity of clinicians working as educators in health professional programs (medicine, paramedics, and now dentistry). In 2018 she received a Senior Fellowship of the UK Higher Education Academy.
