# Supplementary material for: 'Compassion, the first emotion ditched when I’m busy’. The struggle to maintain our common humanity
Source: MedEdPublish (2016). 2018 Aug 14;7:167. [Version 1] doi: 10.15694/mep.2018.0000167.1 (PMC10701822; doi:10.15694/mep.2018.0000167.1)
Supplement: Peer review history

# Reviewer report: Reviewer response for version 1

**DOI:** 10.21956/mep.19632.r29230

**Published:** 2018-08-24

**Authors:** Jayne Garner

**Affiliations:** 1 university of liverpool

This review has been migrated. The reviewer awarded 4 stars out of 5 This paper is very well presented, providing solid evidence and methodological context. What I found most useful were the actual quotes, lending the participants a clear voice, alongside the author recommendations. When reading the paper, I was thinking about the current literature on ‘burn out’ and resident depression, and how this work offers a more positive/practical focus to a very difficult transition period. Thank you.

Reviewer Expertise:

NA

No decision status is available

# Reviewer report: Reviewer response for version 1

**DOI:** 10.21956/mep.19632.r29233

**Published:** 2018-08-17

**Authors:** Victoria Brazil

**Affiliations:** 1 Bond University

This review has been migrated. The reviewer awarded 4 stars out of 5 Thanks for a fascinating deep dive into an important issue.I'm going to offer more appreciation than critique.While a masterclass in qualitative methods, its also expressed beautifully for clinician audience with less experience of those methods.The recommendations make sense, and i wonder how best we can disseminate them more widely to the junior doctors themselves?

Reviewer Expertise:

NA

No decision status is available

# Reviewer report: Reviewer response for version 1

**DOI:** 10.21956/mep.19632.r29227

**Published:** 2018-08-17

**Authors:** David Taylor

**Affiliations:** 1 Gulf Medical University, Ajman, UAE

This review has been migrated. The reviewer awarded 5 stars out of 5 I thoroughly enjoyed reading this paper, and particularly the way in which the lived experience of the participants were analysed. Clearly in condensing the doctorial study into a research paper some of the nuances will be lost, but this is exceptionally well written and I found myself stopping and thinking after nearly every quote. The take home message for me relates to the self-efficacy theme and the danger of conflating clinical competence with self-worth.I shall be recommending this paper to all of my students – both research and clinical!

Reviewer Expertise:

NA

No decision status is available

# Reviewer report: Reviewer response for version 1

**DOI:** 10.21956/mep.19632.r29231

**Published:** 2018-08-15

**Authors:** Gulshat Kemelova

**Affiliations:** 1 Karaganda State Medical University

This review has been migrated. The reviewer awarded 3 stars out of 5 This is a interesting article of a well-executed study on an important topic about empathy and compassion to patients. The discussion and recommendation sections in particular were well-written. Doctor's diligence may be different. Some of doctors can exercise it reluctantly, not because of the desire to help, but because it seems to them: "We have no choice and this is our job". A feeling of compassion or empathy is an internal state and it is negative emotions because of the problems of other people. Of course, doctors should be able to feel compassion for patients and understand the feelings of others in order to establish a strong relationship. It is important for every patient to be aware of his support. However, one should not unduly sink into the suffering of other people, it is important to be able to stop and not harm youself by others' misfortunes, everything should be within reasonable limits. Of course, the problem discussed in the article is interesting, but it is not completely uncovered. In the methods of study is not possible to determine how it is possible to track the progress in the negative or positive side of the ability to develop empathy of interns, with what are type of patients they worked, what was the outcome in patients, because different types of patients by age and diagnosis express different types of problems, and accordingly there may be different levels of empathy. In my opinion, this article does not clearly reveal the problem of developing empathy for interns.It is unclear, based on what data are given recommendations for subsequent practice. Also, I would like to note that the title of the article does not correspond to what is described in this article.

Reviewer Expertise:

NA

No decision status is available

# Reviewer report: Reviewer response for version 1

**DOI:** 10.21956/mep.19632.r29228

**Published:** 2018-08-15

**Authors:** Gary D. Rogers

**Affiliations:** 1 Griffith University

This review has been migrated. The reviewer awarded 3 stars out of 5 This is a thoughtful and interesting report of a soundly-executed study on an important topic. The discussion section in particular was exquisitely well-written. The authors provided some justification for their decision to utilise an amalgam of analytical techniques rather that one of the well-developed methodologies such as Smith’s Interpretative Phenomenological Analysis or Giorgi’s Descriptive Phenomenology, but I would have liked to have understood more about this rather controversial choice. The themes in the findings were nicely encapsulated but it would have been good to see a little more quotation-based support for each of them, perhaps in a table to avoid lengthening the paper unduly. A couple of important points did not come out in the discussion and the authors may want to consider addressing these if they revise the paper after feedback. The first is the meaning of the clear developmental trajectories that they have documented vis a vis the so-called ‘empathy paradox’. This is a debate that has emerged from the completely inconsistent findings of (mostly quantitative) studies of ‘empathy’ in relation to whether it develops, wanes or stays the same across medical training. The idiographic nature of this study’s findings and the evident contingency of compassion (and thus, probably, empathy) on each respondent’s personal emotional state at any point of time have a lot to say in answer to this ongoing debate. Secondly, I would like to see the authors consider the likely effect that the *process* of journaling and being interviewed might have had on the development of these participants. The authors acknowledge (rightly) that their volunteers may have self-selected for ‘reflectiveness’ (and thus the capacity to develop) but there ought to have been an opportunity to find evidence within the transcripts of where the very process of taking part in facilitated reflection (the study activities) actually had an educational or developmental effect in taking them along the trajectory that the authors have so nicely described. For an educational journal, this is probably the most interesting potential of the study.A couple of quibbles: There were quite a few typographical and formatting problems that detracted from the reading experience – especially inconsistent formatting of the quotes – that it would be valuable to fix in a revision. Additionally, the ‘Take Home Messages’ have been couched as messages for interns whereas these should really be messages for educators for a paper in this journal.I hope the authors do decide to revise and resubmit this paper as it adds significantly to our understanding of a complex and important phenomenon.

Reviewer Expertise:

NA

No decision status is available

# Reviewer report: Reviewer response for version 1

**DOI:** 10.21956/mep.19632.r29232

**Published:** 2018-08-14

**Authors:** Ken Masters

**Affiliations:** 1 Sultan Qaboos University

This review has been migrated. The reviewer awarded 4 stars out of 5 An extremely well-written paper, dealing with a very difficult topic. The authors have situated the research firmly in the literature, have clearly laid out illustrative examples of the interns’ writings, and then have used the Discussion well to return to the literature and reflect upon their findings. All this while dealing sensitively with the material.Although the sample is small, it is enough to identify the complexities involved. A larger sample might merely have indicated a greater complexity. One point on the paper’s structure: I don’t think Table 2 should come in the Conclusion. If the authors submit a revised version of their paper (and I recommended that they do), I would suggest that they move Table 2 into the Discussion. If they do not wish to break the narrative in the Discussion, then perhaps move it to an Appendix, but refer to it in the Discussion, but that may make it difficult for the reader.I would also like to have seen more samples of comments from the interns. I understand that placing too many in the Results may break the narrative, but the authors could use the opportunity of the Appendices or Supplementary files to show more of these.

Reviewer Expertise:

NA

No decision status is available

# Reviewer report: Reviewer response for version 1

**DOI:** 10.21956/mep.19632.r29226

**Published:** 2018-08-14

**Authors:** Trevor Gibbs

**Affiliations:** 1 AMEE

This review has been migrated. The reviewer awarded 5 stars out of 5 A very well-written and well-researched paper that "digs deep" into a very important element of physician development. I enjoyed reading it and valued not only the sound theoretical basis for the writing but the way the research was organised and most importantly how it didn't stop at the problem level but briefly explored the solutions available.It is a pity that more students didn't embark upon the longitudinal study, but this may have a reason all of its own! I think that the well structured qualitative approach overcome the low response rate.I would strongly recommend this paper to all involved in curriculum development, whether at undergraduate or postgraduate level.

Reviewer Expertise:

NA

No decision status is available

# Reviewer report: Reviewer response for version 1

**DOI:** 10.21956/mep.19632.r29229

**Published:** 2018-08-14

**Authors:** Dujeepa D. Samarasekera

**Affiliations:** 1 National University of Singapore

This review has been migrated. The reviewer awarded 4 stars out of 5 Very illuminating paper. Enjoyed and learnt from the paper as well. The paper highlights a very important area in training esp PGY1. This may also shed light on our young doctors' burnout in early clinical practice. The authors also highlight the importance of the need to develop self-reflection and resilience among the doctors and include this as part of training. The table highlighting what the faculty and the supervisors need to focus on is very relevant and useful.

Reviewer Expertise:

NA

No decision status is available
